# Examining the Survey Setting Effect on Current E-Cigarette Use Estimates among High School Students in the 2021 National Youth Tobacco Survey

**DOI:** 10.3390/ijerph19116468

**Published:** 2022-05-26

**Authors:** Julia Chen-Sankey, Michelle T. Bover Manderski, William J. Young, Cristine D. Delnevo

**Affiliations:** 1 Center for Tobacco Studies, Rutgers Biomedical and Health Sciences, New Brunswick, NJ 08901, USA; bovermi@cts.rutgers.edu (M.T.B.M.); william.j.young@rutgers.edu (W.J.Y.); delnevo@cts.rutgers.edu (C.D.D.); 2 School of Public Health, Rutgers University, Piscataway, NJ 08854, USA

**Keywords:** survey setting effect, e-cigarette use, health risk behavior, youth, tobacco use

## Abstract

The 2021 National Youth Tobacco Survey (NYTS) was completed by youth online during class time, either in school or at home due to the COVID-19 pandemic. Given the role of NYTS data in tobacco regulatory science, it is vital to understand the effect of survey settings (home, school) on tobacco-use estimates. We used a series of multivariable logistic regressions to examine whether survey settings (home vs. school) predicted current e-cigarette use among high school students, controlling for other known predictors of e-cigarette use as well as the pandemic learning model that was dominant in students’ counties (e.g., nearly all at-home, majority in school). We observed a significant survey setting effect. Those who completed the survey in school had higher odds of current e-cigarette use than those who completed the same survey at home (AOR = 1.74); this effect was attenuated when we controlled for the pandemic learning model (AOR = 1.38). Moreover, e-cigarette use was independently associated with students’ learning model; students whose schools were nearly entirely in-person had the highest odds of e-cigarette use compared to students whose learning model was nearly all at-home (AOR = 1.65). Survey setting is a methodological artifact in the 2021 NYTS. Perceived privacy and peer effects can potentially explain this artifact.

## 1. Introduction

According to data from the U.S. National Youth Tobacco Survey (NYTS) administered by the U.S. Centers for Disease Control and Prevention (CDC) and the Food and Drug Administration (FDA), youth tobacco use patterns in the United States have changed drastically in the last decade. For example, past-30-day cigarette smoking prevalence among high school students steadily decreased from 15.8% in 2011 to 4.6% in 2020 [1,2,3]. In contrast with cigarette smoking, past-30-day electronic cigarette (or e-cigarette) use among high school students rose substantially between 2011 and 2015, then declined in subsequent years before dramatically increasing to an all-time high of 27.5% in 2019 [1,2]. In 2020, prevalence had declined to 19.6% [3], and the most recent 2021 NYTS data suggest that e-cigarette use prevalence among high school students may have declined once again (11.3%) [3,4]. However, due to challenges arising from the COVID-19 pandemic, the 2021 NYTS was conducted differently than in previous years, and as a result, its sponsors urged caution when comparing 2021 estimates to those from previous years [4].

Prior to 2021, NYTS data collection took place at school using either a paper and pencil format or a tablet-based administration with offline data collection [5]. In 2020, data collection was unexpectedly cut short due to disruption caused by the pandemic [6]. Given continued emergency COVID-19 protocols at schools (e.g., distance and hybrid-learning models, restricted visitor access), the 2021 NYTS was administered as an online survey for the first time, and was completed by youth respondents while physically at school, at home, or somewhere else during a designated class period [5].

Survey science literature indicates that methodological factors such as question wording, survey setting, and survey-administration techniques can lead to differences in survey responses and prevalence estimates [7,8,9,10]. For example, one study found that the “check-all-that-apply” response format cut youth tobacco use prevalence estimates nearly in half compared to the “forced-choice” response format [7]. Other studies found that active parental consent procedures produced lower cigarette-smoking estimates among youth compared to passive parental consent [9,10]. Of direct relevance to the 2021 NYTS, the setting in which data collection takes place (i.e., home vs. school) may affect the accuracy of estimates of youth risk-behavior such as tobacco and drug use [11,12,13,14,15]. Specifically, past research indicates that youth may report higher tobacco use when completing surveys at schools vs. at home [11,12,13], potentially due to increased perceived privacy and peer presence at school than at home [11,16,17,18].

Accurate population measures of tobacco use are critical to understanding the prevalence and patterns of tobacco use and trends among youth, as well as informing youth tobacco prevention and control programs and policies. Given the important role of NYTS data in that process, it is vital to understand how different survey locations in 2021 may have affected estimates for that year. This study, therefore, seeks to quantify those effects on past-30-day (current) e-cigarette use prevalence among high-school students, the group with the highest e-cigarette use prevalence and a priority population for tobacco control [4,19].

The ideal study design to measure the effect of survey setting on past-30-day e-cigarette use prevalence would have been a split-sample experiment in which half of the students in the 2021 NYTS were randomly assigned to complete the survey at school and the other half randomly assigned to complete it at home. However, given the variation in responses to the pandemic at both the school district and individual levels (e.g., some districts offered in-person or hybrid learning, while others did not), survey setting was not randomly assigned, complicating efforts to assess its causal impact. In the absence of a split-sample design, we employ standard statistical methods alongside a quasi-experimental approach that facilitates drawing causal inferences from observational data. In the process, we present estimates of the impact of the survey setting and attempt to disentangle them from the effects of peer influence.

## 2. Materials and Methods

### 2.1. Sample

Data come from the 2021 National Youth Tobacco Survey, a nationally representative survey based on a stratified, three-stage cluster sample design [5]. The 2021 NYTS was administered as an online survey, supported virtually by trained technical assistants. Middle- and high-school students participated in the survey while at school, at home, or, uncommonly, elsewhere during a designated class period as part of a classroom activity. Using a school-issued or personal internet-connected device, students watched a 2 min instructional video before completing the survey. Participation in the NYTS was voluntary at both the school and student levels, and at the student level, participation was anonymous. More information about the 2021 NYTS survey and its administration can be found elsewhere [5]. Because the focus of this study is the prevalence of past-30-day e-cigarette use among high school students, as well as the impact of the school vs. home setting, we restrict our analyses to high-school students who completed the survey either at school or at home (grades 9–12; unweighted *n* = 10,212).

### 2.2. Measures

Demographic covariates assessed in the study included *Sex* (female or male), *Race/ethnicity* (non-Hispanic White, non-Hispanic Black, Hispanic, or non-Hispanic other race), *Grade level* (9th, 10th, 11th, 12th), and *Grades earned in school* (mostly As, mostly Bs, mostly Cs, mostly Ds, mostly Fs, not answered/displayed). *Survey setting* assessed in the study was from a measure that describes the location of survey administration (home, school, and other). We also created a measure representing the pandemic *Learning model* that was dominant in the county of each student. In brief, we examined the distribution of the survey setting for each geographical area (i.e., PSU) and coded each as operating under an exclusively or nearly exclusive at-home model (nearly all at-home model; ≥90% at home), a majority at-home model (60–89% at home), an approximately half at-home and half in-school model (about even model), a majority in-school model (60–89% in school), or an exclusively or nearly exclusive in-school model (nearly all in-school model; ≥90% in school). This measure was used as a proxy to represent the magnitude of peer interaction at the time the survey was administered.

### 2.3. Statistical Analyses

We calculated the distribution of characteristics of the study population overall and by survey setting, as well as the prevalence of past-30-day e-cigarette use by demographics. We then fit a series of multivariable logistic regression models to assess the association between survey setting (in school vs. at home) and past-30-day e-cigarette use, adjusted for sex, race/ethnicity, and grade level. To differentiate between the effect of survey setting itself on e-cigarette use and the impact of being around one’s peers, Model 2 additionally included the learning model. A third model further adjusted for earned academic grades to examine whether the setting and peer-influence effects still hold after controlling for this known predictor of tobacco use among youth [20]. All analyses accounted for the complex sample design using the PSU, strata, and weighting variables provided by NYTS to produce nationally representative estimates. Observations with missing values for analysis variables were excluded from the final analysis during multivariable regression modeling using listwise deletion, except when missingness exceeded 5%, as was the case with school grades. For school grades, *not answered* was treated as a valid category in the analysis. Those who reported “Other” (0.3% of the total sample size) for the survey setting question were removed from the analysis.

Given the non-random assignment of survey setting and the resulting imbalance in predictors of e-cigarette use (Table 1), the models described in the preceding paragraph could produce biased inferences. Therefore, we repeated the above analyses after employing coarsened exact matching to preprocess the data [21]. When combined with familiar inferential techniques, such matching methods (and coarsened exact matching in particular) can facilitate causal inferences in observational studies by restricting analyses to the subset of the data for which there are close “matches” [22,23]. That is, matching approximates a controlled experiment by producing a dataset in which there is little to no imbalance in prespecified covariates between the groups of students who take the survey at school versus at home. To run the matched analyses, we matched on race/ethnicity, sex, grade level, and grades earned in school, which, indeed, resulted in greater balance (pre-match L1: 0.32; post-match L1: 0.17). The weights produced by the matching procedure were multiplied by the existing weights provided by NYTS. Their product was the new weight used in the regression analyses. All other procedures were the same, as stated in the preceding paragraph. Analyses were completed in STATA version 17 (StataCorp, College Station, TX, USA) and SAS software version 9.4 (SAS Institute, Cary, NC, USA).

## 3. Results

Table 1 contains the characteristics of the study population overall and stratified by survey setting. Overall, about half of the students completed the survey at home (52.7% [95% CI 44.7–60.6]) and half did so at school (47.3% [95% CI 39.4–55.3]). There was greater variation in the extent to which students’ districts provided in-school learning (e.g., 21.1% nearly all at-home model, and 19.6% nearly all in-school model). Compared to students who took the survey at home, a far larger proportion of students who completed the survey at school were non-Hispanic White (67.5% [95% CI 62.4–72.5] vs. 36.8% [29.3–44.2]), while a notably smaller proportion were Hispanic (14.0% [CI 9.8–18.1] vs. 35.4% [28.2–42.6]). A somewhat larger proportion of students who took the survey in school were male (56.3% [95% CI 52.36–60.0] vs. 50.5 [47.0–53.9]). Members of this group were also less likely to answer the question about the grades they receive in school than their counterparts at home (8.8% [95% CI 7.0–10.5] vs. 12.7% [10.3–15.1]).

An estimated 11.3% (95% CI 9.7–12.9) of high-school students reported using e-cigarettes at least once in the 30 days leading up to the survey (Table 2). Nearly twice the proportion of students that took the survey at school (15.0% [95% CI 12.7–17.2]) compared to at home (8.2% [95% CI 6.8–9.6]) reported past-30-day e-cigarette use. Prevalence also increased with the proportion of learning taking place in school from 7.4% (95% CI 5.2–9.5) among the nearly all at-home learning model to 17.0% (95% CI 13.8–20.2) among the nearly all in-school learning model.

In all regression models, students who took the survey in school had greater odds of reporting past-30-day e-cigarette use (AORs range from 1.38 to 1.74 for adjusted analysis and 1.29 to 1.79 for matched analysis) than those who took the survey at home. Across the three models, as the learning model and then school grades covariates were added, the odds ratios associated with completing the survey in school attenuated slightly but remained significant. Their associated 95 percent confidence intervals also suggest reasonable precision. A full version of Table 2, including results for covariates, is included as a supplement; in all models, male sex and non-Hispanic white race/ethnicity were associated with decreased odds of e-cigarette use, while increased grade level and poorer grades in school were associated with increased odds of e-cigarette use.

Students in areas where learning took place exclusively in school displayed greater odds of past-30-day e-cigarette use, independent of whether they took the survey in school themselves, compared to those who were in districts where learning took place exclusively at home. For example, even after adjusting for demographics, survey setting, and school grades, belonging to a unit where learning took place majority or exclusively in school was significantly associated with greater odds of reporting past-30-day e-cigarette use (OR 1.52 [95% CI 1.08–2.15] and 1.69 [1.14–2.52], respectively). The matched regression models yielded similar findings (OR 1.82 [95% CI 1.29–2.57] and 2.03 [1.38–2.97], respectively.

## 4. Discussion

Our study revealed compelling evidence of both a setting effect in the assessment of past-30-day (current) e-cigarette use in the 2021 NYTS, as well as some form of peer influence. Specifically, high-school students who completed NYTS questionnaires in school had higher odds of reporting current e-cigarette use than those who completed the same questionnaires at home. The same was true of students residing in locations where learning took place majority or exclusively in school (vs. exclusively at home), independent of where they completed the survey themselves.

These findings are consistent with other studies comparing youth’s reported prevalence of risk behaviors when completing surveys in school and home settings [11,12,13,14,24]. Differences in reported prevalence by location could stem from greater perceived privacy at school than at home [11,16,17], where a parent or guardian might see or overhear students’ responses. Indeed, one study found that youth provided with the greater privacy afforded by automated telephone interviewing, in which questions were answered by pressing numbers on a telephone keypad, were more likely to report cigarette-smoking behavior [14].

Higher prevalence in the school setting could be a function of both response bias because they are in the presence of peers, and/or peer influences that promote use (e.g., peer pressure, increased access to e-cigarettes). Youth e-cigarette use is heavily influenced by the e-cigarette-related perceptions and behavior of their peers [25,26,27], and has been increasingly likely to take place in or around school campuses [28,29]. One recent study found that, among sixth-grade students in urban Texas, on-campus learners had greater odds of reporting e-cigarette use susceptibility and ever using e-cigarettes than remote learners [18]. Previous research has also found that to gain increased status among peers, students might be more likely to report drug use when in their presence [30].

Additionally, while previous research has shown that youth’s perceived anonymity or trust when completing surveys may affect survey responses, and therefore behavior prevalence estimates [31,32], it is important to note that because the 2021 NYTS was administered entirely online and student participation was anonymous in both school and home settings, student respondents may have had greater reason to feel comfortable answering freely during this iteration of the survey.

Based on our findings, it is critical for researchers and policymakers to continue to gain knowledge on the impacts of survey setting on youth’s response to tobacco-use-related questionnaires. Future research that uses self-report survey methods to estimate youth tobacco use prevalence should further consider the potential setting effect when planning surveys. The results generated from the surveys (either those implemented at school or home) to be used for estimating youth’s behavior prevalence should be interpreted with caution. Specifically, the results from surveys taken at home or from a mix of home and school settings may provide lower estimates of e-cigarette use prevalence than survey administered exclusively at school. Therefore, instructions for parents and legal guardians to provide sufficient privacy for students to complete surveys at home may help reduce youth’s concerns of reporting health risk behaviors. Finally, future survey research should consider adding items related to survey setting and mode (e.g., perceived anonymity, perceived privacy, perceived presence of peers and authoritative figures) to help explain whether and how the reporting of risk behaviors may vary by those prominent factors.

This study has several limitations. First, the study only assessed past-30-day e-cigarette use prevalence as the outcome for examining the survey setting effect. It is also important to assess the setting effect on the estimates of using other tobacco products among youth, including combustible tobacco products such as cigarettes and cigars. It is possible to observe a stronger survey setting effect on youth cigarette-smoking outcomes, as cigarette smoking may be considered more stigmatized than e-cigarette use among authoritative figures [33,34], therefore increasing student respondents’ privacy concerns when reporting such behavior. Second, using the study design, we cannot fully disentangle survey setting effects from peer-influence effects. This is especially true because students’ pandemic learning model in their residential county is an ecological measure, which may not be applicable to them individually. Third, the NYTS does not include direct measures of family household income or parental education. Therefore, our multivariable regression modeling did not control for those measures. However, we controlled for academic grades in the full model, which serves as a strong predictor of youth tobacco-use behavior [35,36]. Finally, using our analytical method, we cannot discern whether current e-cigarette use among high school students was underestimated among those who completed the survey at home or overestimated among those who completed the survey at school. Further studies are needed to improve our understanding of this question.

As the COVID-19 pandemic continues to affect the administration of research studies that traditionally occurred at school, it is important to critically assess the effects of study location on youth’s tobacco-use responses. Only in doing so can we hope to gain an accurate understanding of tobacco use estimates and patterns at local and national levels in this new context. Since certain methodological factors, such as survey setting, can alter prevalence estimates of health-risk behaviors, these factors should be considered and controlled for when planning survey administration.

## 5. Conclusions

Using the latest NYTS (2021) data from a nationally representative sample of U.S. high school students, this study found a significant survey setting effect on past-30-day (current) e-cigarette use estimates. Students who completed the survey at school had higher odds of reporting current e-cigarette use than those who completed the survey at home; and those who were in the counties where learning took place mainly or exclusively in school, where they were presumably exposed to more peers, had higher odds of reporting current e-cigarette use than those whose counties implemented an exclusive-at-home learning model. Researchers and policymakers need to consider the impact of survey setting effect on youth tobacco use estimates to better inform tobacco-control programs and policies.

## Figures and Tables

**Table 1:** Characteristics of U.S. high-school students overall and by survey setting (unweighted *n* = 10,212).

	Overall		Survey Setting			
			Home		School	
	%	(95% CI)	%	(95% CI)	%	(95% CI)
**Sex**						
Female	46.8	(43.5, 50.1)	49.5	(46.1, 53.0)	43.7	(40, 47.4)
Male	53.2	(49.9, 56.5)	50.5	(47.0, 53.9)	56.3	(52.6, 60.0)
**Race/Ethnicity**						
Non-Hispanic White	51.3	(45.4, 57.2)	36.8	(29.3, 44.2)	67.5	(62.4, 72.5)
Non-Hispanic Black	12.3	(9.5, 15.0)	15.0	(10.8, 19.2)	9.2	(6.7, 11.7)
Hispanic	25.3	(20.7, 29.8)	35.4	(28.2, 42.6)	14.0	(9.8, 18.1)
Non-Hispanic other race	11.2	(8.9, 13.4)	12.8	(9.5, 16.1)	9.4	(7.1, 11.7)
**School Grade**						
9th	26.7	(24.0, 29.3)	25.6	(22.6, 28.6)	27.9	(23.4, 32.4)
10th	25.4	(23.3, 27.4)	25.8	(22.5, 29.1)	24.9	(22.3, 27.6)
11th	24.4	(22.7, 26.1)	24.8	(22.3, 27.4)	23.9	(21.9, 25.9)
12th	23.6	(21.5, 25.6)	23.8	(21.3, 26.3)	23.3	(19.9, 26.7)
**Grades Earned**						
Mostly As	41.7	(38.3, 45.2)	41.3	(36.6, 45.9)	42.2	(38.1, 46.4)
Mostly Bs	27.6	(25.8, 29.4)	25.7	(23.4, 28.1)	29.6	(27.1, 32.0)
Mostly Cs	12.1	(10.6, 13.7)	11.5	(9.3, 13.6)	12.9	(10.9, 14.8)
Mostly Ds	3.9	(3.3, 4.6)	3.9	(3.2, 4.7)	4.0	(3.2, 4.7)
Mostly Fs	3.8	(2.7, 4.8)	4.9	(3.2, 6.6)	2.6	(1.8, 3.3)
Not answered/displayed	10.8	(9.2, 12.4)	12.7	(10.3, 15.1)	8.8	(7.0, 10.5)
**Survey Setting**						
Home	52.7	(44.7, 60.6)				
School	47.3	(39.4, 55.3)				
**Learning Model**						
Nearly all at-home	21.1	(8.8, 33.4)				
Majority at-home	19.2	(6.5, 31.9)				
About even	17.5	(7.7, 27.3)				
Majority in-school	22.7	(10.8, 34.5)				
Nearly all in-school	19.6	(10.7, 28.4)				

NOTE: Percentages are weighted to be representative of U.S. high-school students; confidence intervals (CI) were estimated using Taylor series linearization, accounting for the complex sampling design.

**Table 2:** Adjusted odds of past-30-day e-cigarette use among high-school youth in the US, 2021 NYTS.

			Model 1				Model 2				Model 3			
	Prevalence		Adjusted (*n* = 9907)		Matched (*n* = 9941)		Adjusted (*n* = 9907)		Matched (*n* = 9941)		Adjusted (*n* = 9343)		Matched (*n* = 9377)	
	%	(95% CI)	OR	(95% CI)	OR	(95% CI)	OR	(95% CI)	OR	(95% CI)	OR	(95% CI)	OR	(95% CI)
**Survey setting**														
Home	8.2	(6.8, 9.6)	1.00	-	1.00	-	1.00	-	1.00	-	1.00	-	1.00	-
School	15.0	(12.7, 17.2)	1.74	**(1.40, 2.17)**	**1.79**	**(1.46, 2.20)**	**1.38**	**(1.08, 1.77)**	**1.36**	**(1.08, 1.72)**	**1.40**	**(1.10, 1.77)**	**1.29**	**(1.03, 1.62)**
**Learning model**														
Nearly all at-home	7.4	(5.2, 9.5)					1.00	-	1.00	-	1.00	-	1.00	-
Majority at-home	8.0	(4.7, 11.2)					1.02	(0.62, 1.66)	1.13	(0.67, 1.89)	0.96	(0.58, 1.59)	1.09	(0.66, 1.81)
About even	11.0	(6.1, 15.8)					1.24	(0.76, 2.02)	1.40	(0.85, 2.29)	1.22	(0.75, 1.98)	1.43	(0.87, 2.35)
Majority in-school	13.2	(9.8, 16.6)					**1.46**	**(1.00, 2.12)**	**1.68**	**(1.16, 2.44)**	**1.52**	**(1.08, 2.15)**	**1.82**	**(1.29, 2.57)**
Nearly all in-school	17.0	(13.8, 20.2)					**1.65**	**(1.10, 2.47)**	**1.89**	**(1.30, 2.75)**	**1.69**	**(1.14, 2.52)**	**2.03**	**(1.38, 2.97)**
**Overall**	11.3	(9.7, 12.9)												

Note: Models 1 and 2 adjusted for sex, race/ethnicity, grade level; Model 3 adjusted for covariates in Models 1–2 and grades earned in school. Full model results available as Supplementary Table S1. CI—confidence Interval; OR—odds ratio; percentages are weighted to be representative of U.S. high-school students; variance was estimated using Taylor Series linearization, accounting for the complex sampling design.

## Data Availability

Data are available on the CDC’s National Youth Tobacco Survey website: https://www.cdc.gov/tobacco/data_statistics/surveys/nyts/data/index.html (accessed on 1 April 2022).

## Supplementary Materials

The following supporting information can be downloaded at: https://www.mdpi.com/article/10.3390/ijerph19116468/s1, Table S1: Adjusted Odds of Past-30-Day E-cigarette Use among High School Youth in the US, 2021 NYTS (full model results).
