# Supplementary material for: Examining the Survey Setting Effect on Current E-Cigarette Use Estimates among High School Students in the 2021 National Youth Tobacco Survey
Source: Int J Environ Res Public Health. 2022 May 26;19(11):6468. doi: 10.3390/ijerph19116468 (PMC9180474; doi:10.3390/ijerph19116468)
Supplement: Supplementary file 1 [file ijerph-19-06468-s001.zip › ijerph-1717344-supplementary.pdf]

Table S1. Adjusted Odds of Past-30-Day E-cigarette Use Among High School Youth in the US, 2021 NYTS (full model results)

				Model 1						Model 2						Model 3					
	Prevalence			Adjusted (N=9907)			Matched (N=9941)			Adjusted (N=9907)			Matched (N=9941)			Adjusted (N=9343)			Matched (N=9377)		
	%	95% CI		OR	95% CI		OR	95% CI		OR	95% CI		OR	95% CI		OR	95% CI		OR	95% CI	
**Sex**																					
Female	11.9	10.0	13.9	1.00	-	-	1.00	-	-	1.00	-	-	1.00	-	-	1.00	-	-	1.00	-	-
Male	10.7	8.9	12.4	**0.82**	**0.69**	**0.99**	**0.82**	**0.68**	**1.00**	**0.83**	**0.69**	**0.99**	0.83	0.68	1.00	**0.70**	**0.57**	**0.85**	**0.69**	**0.56**	**0.85**
**Race/Ethnicity**																					
Non-Hispanic White	14.3	12.2	16.4	1.00	-	-	1.00	-	-	1.00	-	-	1.00	-	-	1.00	-	-	1.00	-	-
Non-Hispanic Black	5.7	3.6	7.8	**0.43**	**0.28**	**0.68**	**0.46**	**0.29**	**0.73**	**0.43**	**0.28**	**0.68**	**0.45**	**0.28**	**0.73**	**0.34**	**0.21**	**0.55**	**0.36**	**0.22**	**0.59**
Hispanic	7.7	5.8	9.5	**0.60**	**0.47**	**0.77**	**0.74**	**0.58**	**0.96**	**0.65**	**0.52**	**0.81**	0.81	0.64	1.04	**0.53**	**0.42**	**0.68**	**0.65**	**0.50**	**0.84**
Non-Hispanic other race	11.4	8.8	14.1	0.87	0.68	1.10	0.87	0.67	1.13	0.90	0.70	1.16	0.91	0.69	1.20	0.87	0.67	1.12	0.90	0.68	1.19
**Grade**																					
9th	6.6	5.0	8.2	1.00	-	-	1.00	-	-	1.00	-	-	1.00	-	-	1.00	-	-	1.00	-	-
10th	9.3	6.9	11.6	**1.53**	**1.12**	**2.09**	**1.68**	**1.19**	**2.36**	**1.54**	**1.12**	**2.10**	**1.68**	**1.19**	**2.36**	**1.56**	**1.14**	**2.14**	**1.69**	**1.20**	**2.38**
11th	13.4	10.9	15.9	**2.29**	**1.71**	**3.05**	**2.46**	**1.80**	**3.36**	**2.31**	**1.73**	**3.08**	**2.48**	**1.82**	**3.38**	**2.43**	**1.83**	**3.24**	**2.70**	**1.98**	**3.68**
12th	16.2	13.6	18.9	**2.85**	**2.10**	**3.87**	**3.01**	**2.18**	**4.17**	**2.86**	**2.09**	**3.90**	**3.03**	**2.18**	**4.21**	**3.07**	**2.26**	**4.17**	**3.35**	**2.42**	**4.64**
**Survey Setting**																					
Home	8.2	6.8	9.6	1.00	-	-	1.00	-	-	1.00	-	-	1.00	-	-	1.00	-	-	1.00	-	-
School	15.0	12.7	17.2	**1.74**	**1.40**	**2.17**	**1.79**	**1.46**	**2.20**	**1.38**	**1.08**	**1.77**	**1.36**	**1.08**	**1.72**	**1.40**	**1.10**	**1.77**	**1.29**	**1.03**	**1.62**
**Learning Model**																					
Nearly all at-home	7.4	5.2	9.5							1.00	-	-	1.00	-	-	1.00	-	-	1.00	-	-
Majority at-home	8.0	4.7	11.2							1.02	0.62	1.66	1.13	0.67	1.89	0.96	0.58	1.59	1.09	0.66	1.81
About even	11.0	6.1	15.8							1.24	0.76	2.02	1.40	0.85	2.29	1.22	0.75	1.98	1.43	0.87	2.35
Majority in-school	13.2	9.8	16.6							**1.46**	**1.00**	**2.12**	**1.68**	**1.16**	**2.44**	**1.52**	**1.08**	**2.15**	**1.82**	**1.29**	**2.57**
Nearly all in-school	17.0	13.8	20.2							**1.65**	**1.10**	**2.47**	**1.89**	**1.30**	**2.75**	**1.69**	**1.14**	**2.52**	**2.03**	**1.38**	**2.97**
**School Grades**																					
Mostly A's	7.5	6.0	8.9													1.00	-	-	1.00	-	-
Mostly B's	11.2	9.3	13.2													**1.89**	**1.52**	**2.34**	**1.96**	**1.56**	**2.45**
Mostly C's	14.6	11.2	18.0													**2.83**	**2.21**	**3.62**	**2.91**	**2.24**	**3.78**
Mostly D's	15.5	10.3	20.6													**3.07**	**1.93**	**4.89**	**3.02**	**1.77**	**5.15**
Mostly F's	16.1	10.3	21.9													**4.06**	**2.80**	**5.89**	**3.69**	**2.45**	**5.57**
No answer/ Displayed	22.6	17.3	27.8													**4.82**	**3.57**	**6.52**	**5.33**	**3.84**	**7.38**

CI, Confidence Interval; OR, Odds Ratio;

Percentages are weighted to be representative of US high school students; Variance was estimated using Taylor Series Linearization, accounting for the complex sampling design
